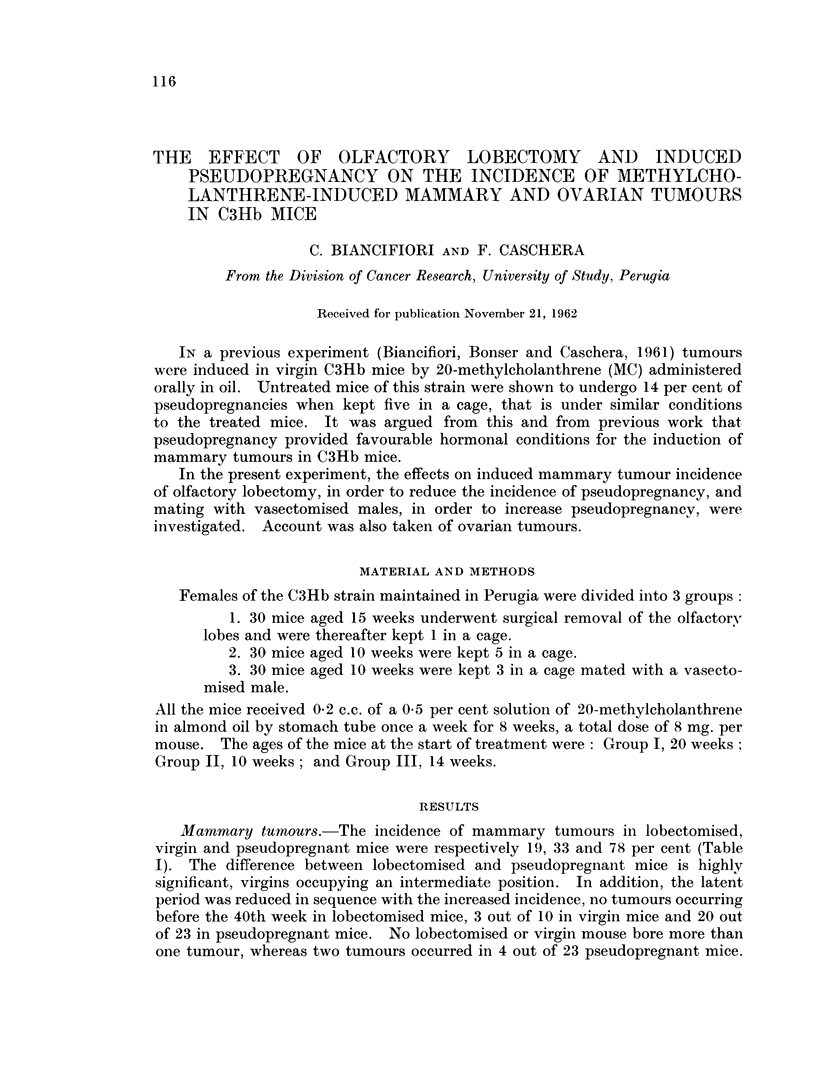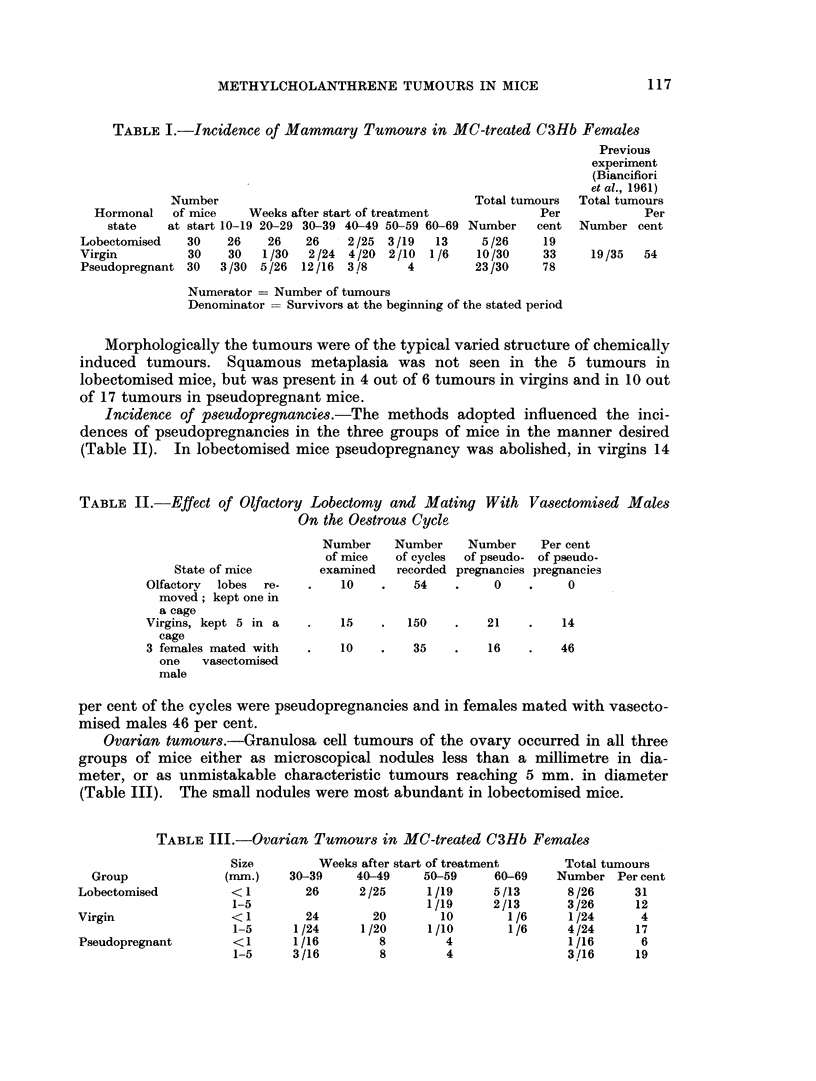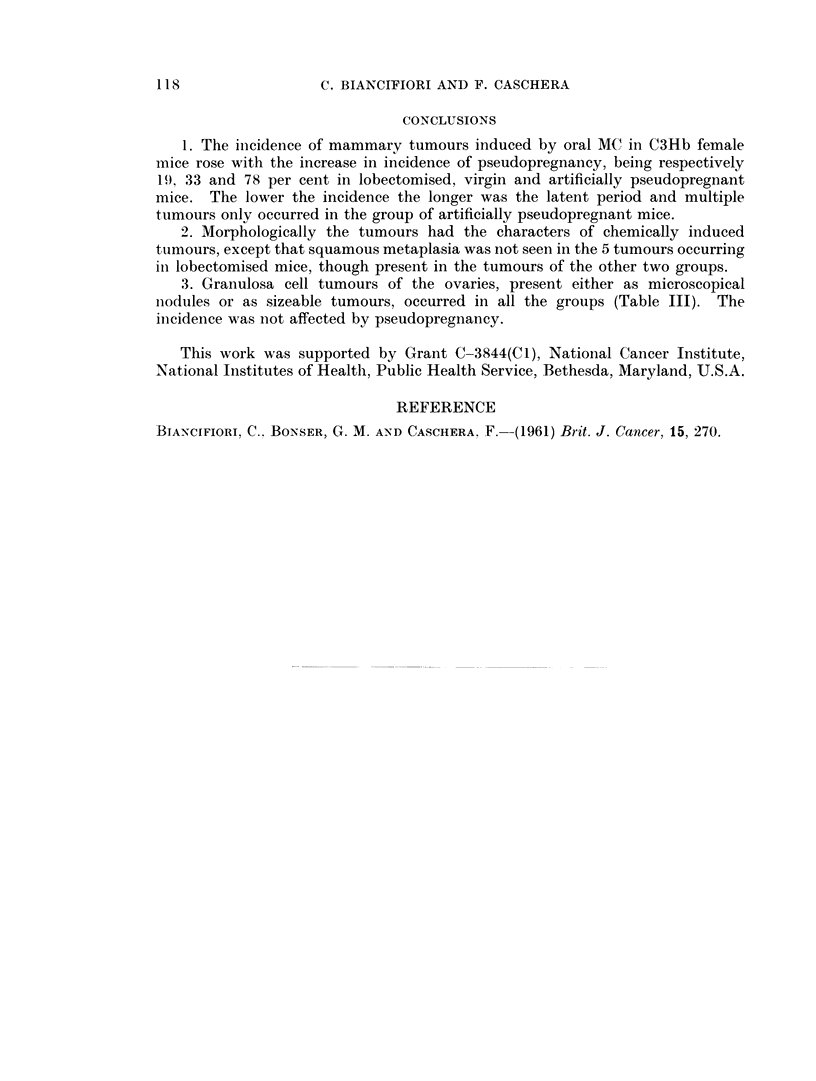# The Effect of Olfactory Lobectomy and Induced Pseudopregnancy on the Incidence of Methylcholanthrene-induced Mammary and Ovarian Tumours in C3Hb Mice

**DOI:** 10.1038/bjc.1963.17

**Published:** 1963-03

**Authors:** C. Biancifiori, F. Caschera


### THE EFFECT OF OLFACTORY LOBECTOMY AND INDUCED PSEUDOPREGNANCY ON THE INCIDENCE OF METHYLCHOLANTHRENE-INDUCED MAMMARY AND OVARIAN TUMOURS IN C3Hb MICE

C. BIANCIFIORI AND F. CASCHERA

*From the Division of Cancer Research, University of Study, Perugia*

Received for publication November 21, 1962

IN a previous experiment (Biancifiori, Bonser and Caschera, 1961) tumours were induced in virgin C3Hb mice by 20-methylcholanthrene (MC) administered orally in oil. Untreated mice of this strain were shown to undergo 14 per cent of pseudopregnancies when kept five in a cage, that is under similar conditions to the treated mice. It was argued from this and from previous work that pseudopregnancy provided favourable hormonal conditions for the induction of mammary tumours in C3Hb mice.

In the present experiment, the effects on induced mammary tumour incidence of olfactory lobectomy, in order to reduce the incidence of pseudopregnancy, and mating with vasectomised males, in order to increase pseudopregnancy, were investigated. Account was also taken of ovarian tumours.

### MATERIAL AND METHODS

Females of the C3Hb strain maintained in Perugia were divided into 3 groups :

1. 30 mice aged 15 weeks underwent surgical removal of the olfactory lobes and were thereafter kept 1 in a cage.
2. 30 mice aged 10 weeks were kept 5 in a cage.
3. 30 mice aged 10 weeks were kept 3 in a cage mated with a vasectomised male.

All the mice received 0·2 c.c. of a 0·5 per cent solution of 20-methylcholanthrene in almond oil by stomach tube once a week for 8 weeks, a total dose of 8 mg. per mouse. The ages of the mice at the start of treatment were : Group I, 20 weeks ; Group II, 10 weeks ; and Group III, 14 weeks.

### RESULTS

*Mammary tumours.*—The incidence of mammary tumours in lobectomised, virgin and pseudopregnant mice were respectively 19, 33 and 78 per cent (Table I). The difference between lobectomised and pseudopregnant mice is highly significant, virgins occupying an intermediate position. In addition, the latent period was reduced in sequence with the increased incidence, no tumours occurring before the 40th week in lobectomised mice, 3 out of 10 in virgin mice and 20 out of 23 in pseudopregnant mice. No lobectomised or virgin mouse bore more than one tumour, whereas two tumours occurred in 4 out of 23 pseudopregnant mice.

TABLE I.—*Incidence of Mammary Tumours in MC-treated C3Hb Females*

| Hormonal state | Number of mice at start | Weeks after start of treatment 10–19 | 20–29 | 30–39 | 40–49 | 50–59 | 60–69 | Total tumours Number | Per cent | Previous experiment (Biancifiori *et al.*, 1961) Total tumours Number | Per cent |
|---|---|---|---|---|---|---|---|---|---|---|---|
| Lobectomised | 30 | 26 | 26 | 26 | 2/25 | 3/19 | 13 | 5/26 | 19 | | |
| Virgin | 30 | 30 | 1/30 | 2/24 | 4/20 | 2/10 | 1/6 | 10/30 | 33 | 19/35 | 54 |
| Pseudopregnant | 30 | 3/30 | 5/26 | 12/16 | 3/8 | 4 | | 23/30 | 78 | | |

Numerator = Number of tumours
Denominator = Survivors at the beginning of the stated period

Morphologically the tumours were of the typical varied structure of chemically induced tumours. Squamous metaplasia was not seen in the 5 tumours in lobectomised mice, but was present in 4 out of 6 tumours in virgins and in 10 out of 17 tumours in pseudopregnant mice.

*Incidence of pseudopregnancies.*—The methods adopted influenced the incidences of pseudopregnancies in the three groups of mice in the manner desired (Table II). In lobectomised mice pseudopregnancy was abolished, in virgins 14 per cent of the cycles were pseudopregnancies and in females mated with vasectomised males 46 per cent.

TABLE II.—*Effect of Olfactory Lobectomy and Mating With Vasectomised Males On the Oestrous Cycle*

| State of mice | Number of mice examined | Number of cycles recorded | Number of pseudo-pregnancies | Per cent of pseudo-pregnancies |
|---|---|---|---|---|
| Olfactory lobes removed; kept one in a cage | 10 | 54 | 0 | 0 |
| Virgins, kept 5 in a cage | 15 | 150 | 21 | 14 |
| 3 females mated with one vasectomised male | 10 | 35 | 16 | 46 |

*Ovarian tumours.*—Granulosa cell tumours of the ovary occurred in all three groups of mice either as microscopical nodules less than a millimetre in diameter, or as unmistakable characteristic tumours reaching 5 mm. in diameter (Table III). The small nodules were most abundant in lobectomised mice.

TABLE III.—*Ovarian Tumours in MC-treated C3Hb Females*

| Group | Size (mm.) | Weeks after start of treatment 30–39 | 40–49 | 50–59 | 60–69 | Total tumours Number | Per cent |
|---|---|---|---|---|---|---|---|
| Lobectomised | $<1$ | 26 | 2/25 | 1/19 | 5/13 | 8/26 | 31 |
| | 1–5 | | | 1/19 | 2/13 | 3/26 | 12 |
| Virgin | $<1$ | 24 | 20 | 10 | 1/6 | 1/24 | 4 |
| | 1–5 | 1/24 | 1/20 | 1/10 | 1/6 | 4/24 | 17 |
| Pseudopregnant | $<1$ | 1/16 | 8 | 4 | | 1/16 | 6 |
| | 1–5 | 3/16 | 8 | 4 | | 3/16 | 19 |

### CONCLUSIONS

1. The incidence of mammary tumours induced by oral MC in C3Hb female mice rose with the increase in incidence of pseudopregnancy, being respectively 19, 33 and 78 per cent in lobectomised, virgin and artificially pseudopregnant mice. The lower the incidence the longer was the latent period and multiple tumours only occurred in the group of artificially pseudopregnant mice.

2. Morphologically the tumours had the characters of chemically induced tumours, except that squamous metaplasia was not seen in the 5 tumours occurring in lobectomised mice, though present in the tumours of the other two groups.

3. Granulosa cell tumours of the ovaries, present either as microscopical nodules or as sizeable tumours, occurred in all the groups (Table III). The incidence was not affected by pseudopregnancy.

This work was supported by Grant C–3844(C1), National Cancer Institute, National Institutes of Health, Public Health Service, Bethesda, Maryland, U.S.A.

### REFERENCE

BIANCIFIORI, C., BONSER, G. M. AND CASCHERA, F.—(1961) *Brit. J. Cancer*, **15**, 270.